# Associated Factors and Prevention of Upper Pole Rippling in Prepectoral Direct-to-Implant Breast Reconstruction

**DOI:** 10.1055/a-2125-7322

**Published:** 2023-11-30

**Authors:** Da Hye Ryu, Oh Young Joo, Yun Ho Roh, Eun Jung Yang, Seung Yong Song, Dong Won Lee

**Affiliations:** 1 Department of Plastic and Reconstructive Surgery, Yonsei University College of Medicine, Seoul, Republic of Korea; 2 Biostatistics Collaboration Unit, Department of Biomedical Systems Informatics, Yonsei University College of Medicine, Seoul, Republic of Korea

**Keywords:** breast reconstruction, prepectoral breast reconstruction, implant rippling, rippling

## Abstract

**Background** Despite its many advantages, prepectoral breast reconstruction also carries the risk of implant rippling. The recent introduction of partial superior implant coverage using a pectoralis muscle slip in prepectoral direct-to-implant (DTI) breast reconstruction has shown the potential to minimize upper pole rippling. The purpose of this study was to identify factors associated with rippling and the effectiveness of our surgical technique.

**Methods** In total, 156 patients (186 breasts) who underwent prepectoral DTI breast reconstruction between August 2019 and March 2021 were identified retrospectively. Patient data were analyzed from medical records. Univariable and multivariable logistic analyses were performed to contextualize the risks associated with rippling deformity relative to demographic characteristics and other clinical factors. Retrospective propensity-matched analysis was performed to identify the relationship between rippling deformity and the reconstruction method.

**Results** Patients with body mass index (BMI; odds ratio [OR], 0.736; *p* < 0.001), those with a postoperative chemotherapy history (OR, 0.324; *p* = 0.027) and those who received breast reconstruction via the superior coverage technique (OR, 0.2; *p* = 0.004), were less likely to develop rippling deformity. The median follow-up period was 64.9 weeks, and there were no significant differences between patients in types of mastectomy, implant, or acellular dermal matrix. Patients who underwent superior coverage technique-based reconstruction showed significantly reduced rippling (OR, 0.083; *p* = 0.017)

**Conclusion** Patients with higher BMI and prior postoperative chemotherapy were less likely to develop rippling deformity. The superior coverage technique can be effective in minimizing upper pole rippling.

## Introduction

Implant based breast reconstruction has become the most commonly practiced method both in the United States and the Republic of Korea. 1 2 There are options that can be considered for implant-based breast reconstruction depending on how it will be inserted. Recently, prepectoral direct-to-implant (DTI) became a more frequently used method with advances in technology and materials, such as intraoperative fluorescence angiography and acellular dermal matrix (ADM). 3 4 5 6

Prepectoral DTI has shown its effectiveness in removing breast animation deformity, alleviating muscle weakness, and limiting postoperative discomfort. As such, it has become an alternative to submuscular implant placement. 7 8 9 10 11 12 13

However, a major drawback of prepectoral implantation compared with the subpectoral method is rippling, which indicates palpable or visible folds on the surface of the reconstructed or augmented breast. As rippling comes with prepectoral DTI despite advantages, patient satisfaction and aesthetic appearance cannot be free from its effects. 14 15 16 A low body mass index (BMI), the performance of revisional surgery, the use of saline implants, and use of textured implants are all risk factors for rippling in augmentation mammoplasty. 17 18 But the study related to breast reconstruction is currently lacking.

In comparison to surgical method trend changes as aforementioned, analysis of rippling factors from immediate prepectoral DTI reconstruction is relatively behind. The study was aimed to understand how different demographics, oncologic treatment, and operative techniques can affect implant rippling for immediate prepectoral DTI.

## Methods

This retrospective study was approved by our facility's institutional review board (no. 4-2022-0946). Written informed consent was obtained from all patients for preoperative and postoperative photography. Consecutive breast cancer patients with ≥1 year of follow-up who underwent prepectoral DTI breast reconstruction in a single center performed by a single plastic surgeon (L. D. W.) between August 2019 and March 2021 were included in this study. Delayed and delayed-immediate cases were excluded. Data pertaining to patient demographics, mastectomy type, axillary lymph node dissection, oncologic stage, implant/ADM variations, application of superior coverage technique, need for either preoperative or postoperative adjuvant therapy, implant type, and aesthetic outcomes were collected ( Table 1 ). Procedural components were decided at the discretion of the treating physician. The superior coverage technique has been performed in patients with prepectoral DTI since December 2020 in our study institution.

**Table 1:** Patient demographics and oncologic characteristics

	Value (%)
Number of breasts	186
Number of patients	156
Age, years	
Median	44
IQR	40.0–51.0
BMI	
Median	22.9
IQR	20.4–25.0
BMI ≥ 25 kg/m ^2^	46 (24.7)
Diabetes mellitus	5 (2.7)
Active smoker	2 (1.1)
Prior RT	0
Prior CT	20 (10.8)
Postoperative RT	33 (17.7)
Postoperative CT	45 (24.2)
Hormone therapy	111 (59.7)
Cancer stage	
0	57 (30.6)
IA/IB	67 (36.0)
IIA/IIB	58 (31.2)
IIIA/IIIB/IIIC	4 (2.2)
IV	0
Follow-up length, weeks	
Median	64.9
IQR	40.0–83.1

Abbreviations: BMI, body mass index; CT, chemotherapy; IQR, interquartile range; RT, radiation therapy.

Preoperative breast volume was measured using a three-dimensional scanner. 19 Follow-up assessments were performed at 1, 3, and 6 months and 1 year after the initial operation and then annually thereafter for the duration of the study. A breast examination was conducted at each follow-up visit to evaluate the development or progression of rippling, implant malposition, capsular contracture, and other complications such as infection. In this study, rippling was defined as visible implant rippling while the patient was in a standing position, and rippling revealed during body bending was excluded. Each postoperative physical examination was performed by a single reviewer (L. D. W.).

### Surgical Technique

Whether or not to perform prepectoral DTI was decided through an evaluation of adequate perfusion of the mastectomy skin flaps following the completion of total mastectomy by an oncologic surgery team. A sizer filled with silicone gel with a volume corresponding to the approximate weight of the mastectomy specimen was inserted into the prepectoral plane. Subsequently, indocyanine green angiography (FLUOBEAM; Fluoptics, Grenoble, France) was performed. If sufficient vascular perfusion was maintained in the mastectomy skin flap, then prepectoral DTI was performed. Patients were interviewed in advance, where it was decided whether to conduct prosthesis-based breast reconstruction.

Regardless of the technique, all implants were inserted with complete implant wrapping with ADM. All implants used in this study were either Mentor (Mentor Worldwide LLC, Santa Barbara, CA,) or BellaGel (HansBiomed Co. Ltd., Seoul, Korea) products, which are both smooth, round-type cohesive silicone gel-filled breast implants. In addition, we used three types of ADMs in this study, Megaderm (L&C BIO Inc., Seongnam, Korea), CGCryoDerm (CGBio Co., Seongnam, Korea), and DermACELL (Stryker Corp., Kalamazoo, MI).

**Fig. 1:**
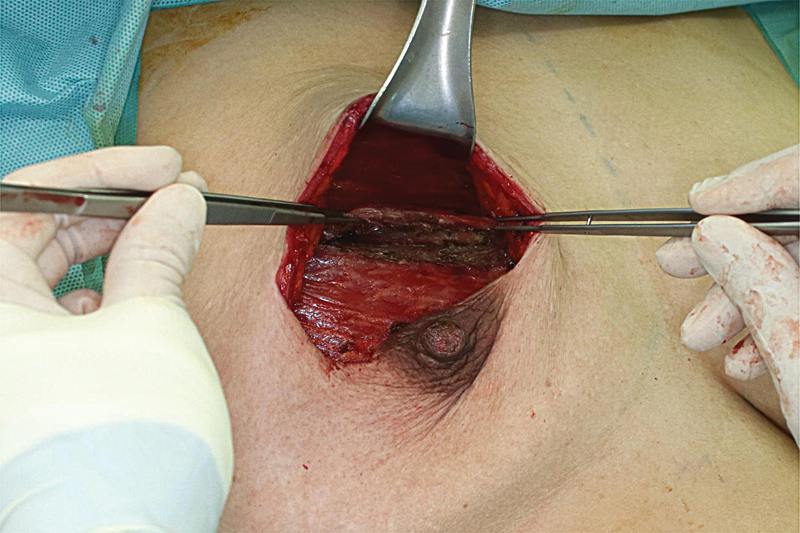
Intraoperative dissection of a pectoralis major muscle slip after the design.

**Fig. 2:**
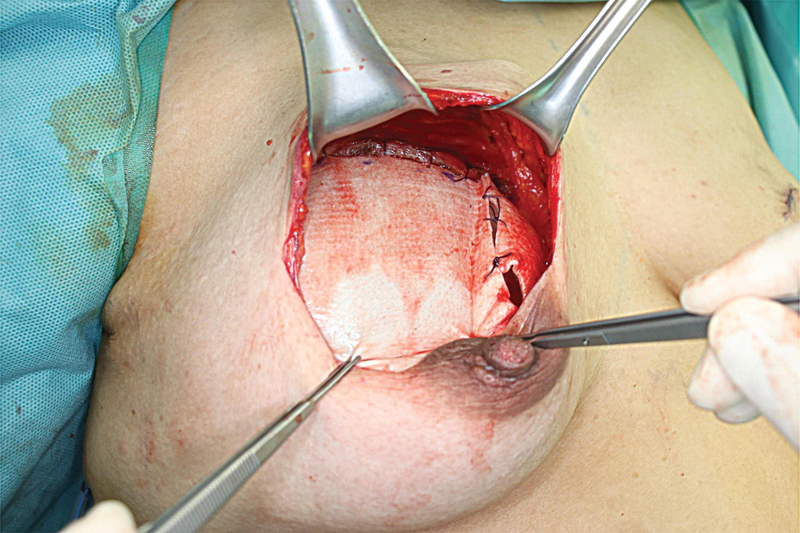
Intraoperative superior coverage of the ADM-wrapped implant by pectoralis muscle slip.

The superior coverage technique in the prepectoral plane was performed with patient customization as follows. After placing the sizer over the pectoralis major muscle and tailor-tacking the incisions, the symmetry relative to the opposite breast was examined with the patient in a sitting position, and perfusion of the mastectomy skin flap was rechecked to avoid disruption caused by the implant. Then, the upper round boundary of the sizer on the pectoralis major muscle was marked, and the sizer was removed. From approximately 1.5 cm below the marked line, an incision was made to be long enough to cover the superior part of the implant, with partial-thickness muscle dissection completed up to create a pectoralis slip ( Fig. 1 ). In addition, incisions were created in the same direction as the muscle belly to minimize muscle function damage. Caution should be taken when dissecting the muscle to avoid damage to the subpectoral fat pad, which contains the thoracoacromial vascular bundle. Also, the muscle must not be disinserted from the sternal origin. After hemostasis was complete, antibiotic irrigation was performed, and two 15-F Blake drains were placed on the inframammary line and the axillary line. After the implant was entirely covered with ADM and placed in the prepectoral plane, the previously created muscle slip was partially placed over the ADM-wrapped implant to cover the superior side. Finally, the ADM was sutured to the inferior edge of the muscle slip with absorbable sutures ( Fig. 2 ). The suture process is performed carefully to avoid damaging the implant and includes placing a malleable retractor through the fenestrated ADM to protect the implant from damage.

The whole procedure was performed using no-touch techniques as much as possible. Prepectoral DTI without the use of a superior coverage technique was performed in the same manner as above except for the creation of a slip of the pectoralis major muscle. The primary outcome was the visibility of any upper pole rippling deformity.

### Statistical Analysis

Univariate logistic regression analysis was performed to calculate odds ratios (ORs) with 95% confidence intervals (CIs) for rippling as a dependent variable. Variables significantly associated with this outcome of interest were input into a multivariable logistic regression model to determine independent predictors of rippling. A propensity score was used to match the group that underwent surgery with the superior coverage technique and the group that did not, which can be thought of as a reliable match between experimental and control groups. A *t* -test (continuous variable) and chi-square test (categorical variable) were used before matching, while a paired *t* -test (continuous variable) and McNemar's test (categorical variable) were used after matching. After reliable propensity score matching, a conditional logistic regression with rippling as a dependent variable in both groups was applied. Statistical significance was set at *p* < 0.05. All statistical analyses were performed using IBM SPSS (IBM Corp., Armonk, NY).

## Results

A total of 156 eligible patients (186 breasts) was identified during the study period ( Table 1 ). The average patient age was 44 years, and the average BMI was 22.9 kg/m ^2^ . Twenty patients (10.8%) had a history of neoadjuvant chemotherapy, 45 patients (24.2%) had received adjuvant chemotherapy, and 33 patients (17.7%) had received adjuvant radiation therapy. A total of 111 patients (59.7%) had received postoperative hormone therapy. The average follow-up length was 64.9 weeks.

**Fig. 3:**
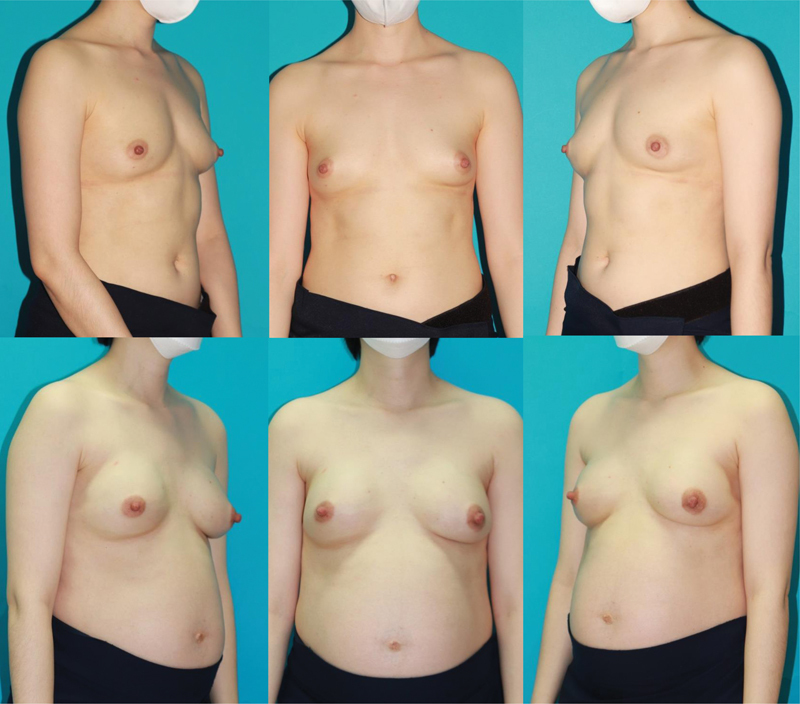
A 32-year-old patient after a bilateral robot assisted nipple-sparing mastectomy through a logitudinal midaxillary line approach for invasive ductal carcinoma (pT1pN0M0) of the right breast and a prophylactic nipple-sparing mastectomy of the left breast after immediate prepectoral DTI with the superior coverage technique. (above: preoperative appearance; below: postoperative appearance at 1 year).

Nipple-sparing mastectomy was performed most commonly, in 158 cases (84.9%), followed by skin-sparing mastectomy (12.4%) and total mastectomy (2.7%; Table 2 ). Five breast surgeons were involved throughout the study period without any differences in the mastectomy technique. Patients were treated with one of two implants (Mentor, 83.3%; BellaGel, 16.7%) and one of three ADMs (Megaderm, 61.8%; CGCryoDerm, 33.9%; and DermACELL, 4.3%). Mastectomy flap necrosis occurred in 15 cases (8.1%), infection occurred in 4 cases (2.2%), and seroma/hematoma occurred in 6 cases (3.2%) and required secondary operative intervention of the skin debridement/drain insertion/implant change ( Fig. 3 ).

**Table 2:** Operative characteristics

	Value (%)
Mastectomy type	
Nipple-sparing mastectomy	158 (84.9)
Skin-sparing mastectomy	23 (12.4)
Total mastectomy	5 (2.7)
Implant type	
Mentor	155 (83.3)
BellaGel	31 (16.7)
ADM type	
Megaderm	115 (61.8)
CGCryoDerm	63 (33.9)
DermACELL	8 (4.3)
ADM thickness	
1.5–2.3 mm	133 (71.5)
1.0–2.0 mm	53 (28.5)
Rippling	44 (23.7)
Superior coverage technique	51 (27.4)
Complication	
Mastectomy skin flap necrosis	15 (8.1)
Infection	4 (2.2)
Implant explantation	2 (1.1)
Seroma/hematoma	6 (3.2)

Abbreviation: ADM, acellular dermal matrix.

The variables associated with rippling were analyzed in a univariate regression analysis ( Table 3 ). A BMI ( *p <* 0.001), postoperative chemotherapy ( *p* = 0.027), and superior coverage technique ( *p* = 0.004) were significantly associated with a decreased risk of rippling. Also, those who underwent skin-sparing mastectomy ( *p* = 0.051) showed a tendency toward less rippling compared with those who underwent nipple-sparing mastectomy.

**Table 3:** Univariate logistic regression for occurrence of implant rippling

Variable	OR	95% CI	*p* -Value
Age	0.979	0.943–1.018	0.288
BMI	0.736	0.653–0.854	<0.001 a
Diabetes	0	0	0.999
Active smoker	3.357	0.206–54.830	0.395
Prior RT	0	0	0.999
Prior CT	0.325	0.072–1.461	0.143
Postoperative RT	0.837	0.336–2.085	0.702
Postoperative CT	0.324	0.119–0.88	0.027 a
Hormone therapy	1.587	0.775–3.251	0.207
Preoperative breast volume, cc	0.998	0.996–1.001	0.148
Specimen weight, g	0.997	0.995–1.000	0.022
Mastectomy type (NSM) (ref.)			
SSM	0.132	0.017–1.008	0.051
TM	0.69	0.075–6.355	0.744
Implant type (BellaGel) (ref.)			
Mentor	1.763	0.634–4.909	0.277
ADM (Megaderm) (ref.)			
CGCryoDerm	0.478	0.217–1.051	0.066
DermACELL	0.355	0.042–2.999	0.342
ADM thickness (1.5–2.3 mm) (ref.)			
ADM thickness (1.0–2.0 mm)	0.813	0.375–1.761	0.6
Superior coverage technique	0.2	0.068–0592	0.004 a
Mastectomy skin flap necrosis	0.218	0.028–1.705	0.147
Infection	0.933	0.112–10.887	0.933
Seroma/hematoma	1.683	0.298–9.520	0.556
Capsular contracture	N/A		

Abbreviations: ADM, acellular dermal matrix; BMI, body mass index; CI, confidence interval; CT, chemotherapy; NSM, nipple-sparing mastectomy; OR, odds ratio; SSM, skin-sparing mastectomy; TM, total mastectomy; RT, radiation therapy; N/A, unable to estimate due to low frequency.

a Statistically significant.

Multivariable logistic regression analysis demonstrated that patients with BMI (OR, 0.690; 95% CI, 0.574–0.829; *p* < 0.001), postoperative chemotherapy (OR, 0.187; 95% CI, 0.056–0.622; *p* = 0.006), and superior coverage technique (OR, 0.169; 95% CI, 0.050–0.569; *p* = 0.004) were independently associated with decreased risk of rippling ( Table 4 ). There were no significant differences in the type of mastectomy, implant, or ADM or in ADM thickness, postoperative radiotherapy, or hormone therapy.

**Table 4:** Multivariate logistic regression for occurrence of implant rippling

Variable	OR	95% CI	*p* -Value
BMI	0.690	0.574–0.829	<0.001 a
Prior CT	0.523	0.082–3.329	0.493
Postoperative RT	2.680	0.785–9.147	0.116
Postoperative CT	0.187	0.056–0.622	0.006 a
Hormone therapy	1.519	0.633–3.644	0.349
Mastectomy type (NSM) (ref.)			
SSM	0.156	0.014–1.704	0.128
TM	3.030	0.091–100.510	0.535
ADM thickness	0.957	0.372–2.462	0.927
Undergoing superior coverage technique	0.169	0.050–0.569	0.004 a

Abbreviations: ADM, acellular dermal matrix; BMI, body mass index; CI, confidence interval; CT, chemotherapy; NSM, nipple-sparing mastectomy; OR, odds ratio; SSM, skin-sparing mastectomy; TM, total mastectomy; RT, radiation therapy.

a Statistically significant.

A total of 135 breasts (72.6%) was treated by prepectoral DTI without the superior coverage technique (patient mean age, 45.7 ± 9.5 years), while 51 breasts (27.4%) underwent surgery with the superior coverage technique (patient mean age, 46.6 ± 8.9 years; Table 5 ). In the groups matched by propensity score, demographics did not differ meaningfully between treated and untreated patients ( *p* ≥ 0.05), which suggests the existence of reliable matching between experimental and control groups.

**Table 5:** Demographics before and after propensity score matching

Variable	Before matching			After matching		
	Prepectoral DTI	Superior coverage technique	*p* -Value	Prepectoral DTI	Superior coverage technique	*p* -Value
	( *n* = 135)	( *n* = 51)		( *n* = 51)	( *n* = 51)	
Age, years	45.7 ± 9.5	46.6 ± 8.9	0.540	47.6 ± 9.7	46.6 ± 8.9	0.792
BMI			0.271			>0.999
BMI < 25 kg/m ^2^	105 (77.8%)	35 (68.6%)		36 (70.6%)	35 (68.6%)	
BMI ≥ 25 kg/m ^2^	30 (22.2%)	16 (31.4%)		15 (29.4%)	16 (31.4%)	
Mastectomy type			0.013			0.693
NSM	121 (89.6%)	37 (72.5%)		42 (82.4%)	37 (72.5%)	
SSM	11 (8.1%)	12 (23.5%)		7 (13.7%)	12 (23.5%)	
TM	3 (2.2%)	2 (3.9%)		2 (3.9%)	2 (3.9%)	
Cancer stage			0.008			0.859
0	42 (31.1%)	15 (29.4%)		17 (33.3%)	15 (29.4%)	
IA/IB	56 (41.5%)	11 (21.6%)		12 (23.5%)	11 (21.6%)	
IIA/IIB	36 (26.7%)	22 (43.1%)		21 (41.2%)	22 (43.1%)	
IIIA/IIIB/IIIC	1 (0.7%)	3 (5.9%)		1 (2.0%)	3 (5.9%)	
Implant type			<0.001			NA
Mentor	104 (77.0%)	51 (100.0%)		51 (100.0%)	51 (100.0%)	
BellaGel	31 (23.0%)	0 (0.0%)				
ADM			0.055			0.572
Megaderm	83 (61.5%)	32 (62.7%)		32 (62.7%)	32 (62.7%)	
CGCryoDerm	49 (36.3%)	14 (27.5%)		16 (31.4%)	14 (27.5%)	
DermACELL	3 (2.2%)	5 (9.8%)		3 (5.9%)	5 (9.8%)	
ADM_thickness			0.474			>0.999
1.5–2.3 mm	99 (73.3%)	34 (66.7%)		37 (72.5%)	34 (66.7%)	
1.0–2.0 mm	36 (26.7%)	17 (33.3%)		14 (27.5%)	17 (33.3%)	
Prior CT			0.590			0.687
No	122 (90.4%)	44 (86.3%)		47 (92.2%)	44 (86.3%)	
Yes	13 (9.6%)	7 (13.7%)		4 (7.8%)	7 (13.7%)	
Postoperative CT			0.276			>0.999
No	99 (73.3%)	42 (82.4%)		40 (78.4%)	42 (82.4%)	
Yes	36 (26.7%)	9 (17.6%)		11 (21.6%)	9 (17.6%)	
Postoperative RT			0.138			0.727
No	115 (85.2%)	38 (74.5%)		43 (84.3%)	38 (74.5%)	
Yes	20 (14.8%)	13 (25.5%)		8 (15.7%)	13 (25.5%)	
Hormone therapy			0.517			0.832
No	52 (38.5%)	23 (45.1%)		25 (49.0%)	23 (45.1%)	
Yes	83 (61.5%)	28 (54.9%)		26 (51.0%)	28 (54.9%)	

Abbreviations: ADM, acellular dermal matrix; BMI, body mass index; CT, chemotherapy; NSM, nipple-sparing mastectomy; SSM, skin-sparing mastectomy; TM, total mastectomy; RT, radiation therapy.

As we confirmed that two groups (with or without superior coverage technique) were matched well based on the use of the superior coverage technique ( Table 5 ), we evaluated the technique's influence after matching by conducting a conditional logistic regression with rippling as a dependent variable in both groups. After matching, patients who underwent surgery with the superior coverage technique showed significantly reduced rippling (OR, 0.083; 95% CI, 0.011–0.643; *p* < 0.017; Table 6 ).

**Table 6:** Rippling frequency and conditional logistic regression analysis (after propensity score matching) for occurrence of implant rippling

Variable	Before matching		After matching	
	Prepectoral DTI ( *n* = 135)	Superior coverage technique ( *n* = 51)	Prepectoral DTI ( *n* = 51)	Superior coverage technique ( *n* = 51)
Rippling	41 (30.4%)	3 (6%)	16 (31.4%)	3 (6%)
Conditional logistic regression analysis (after matching)		OR	95% CI	*p* -Value
Superior coverage technique		0.083	0.011–0.643	0.017 a

Abbreviations: CI, confidence interval; OR, odds ratio; DTI, direct-to-implant.

a Statistically significant.

## Discussion

The therapeutic implications of breast reconstruction after total mastectomy due to breast cancer have been widely demonstrated. Despite these advantages, implant rippling is one of the most common aesthetic limitations in prepectoral DTI breast reconstruction. 20 21 Rippling is caused by soft tissue deficits after mastectomy and is particularly common in the upper poles and superior implant edges. 22 Although there are some options to prevent rippling through ADM coverage, fat grafting and other techniques are also being used. 18 Our study focuses on the factors that affect rippling in prepectoral implant breast reconstruction and considers the effects of the recently introduced superior coverage technique in DTI breast reconstruction.

The superior coverage technique was introduced by Pittman et al in an effort to reduce rippling deformities after prepectoral breast reconstruction. 10 The P1 method and the superior coverage technique are similar, the difference being that our method creates a muscle slip after complete coverage of the implant. To the best of our knowledge, our study is the first to specifically analyze the factors affecting rippling in all DTI patients with or without superior coverage technique and to demonstrate a decreased risk of rippling with our superior coverage technique. This method proceeds by covering the entire implant with ADM and then fixing it to the already-designed pectoralis muscle slip. Superior coverage technique is different from subpectoral breast implant insertion as it only gets a portion of pectoralis muscle, and animation deformity that can occur from subpectoral placement was not observed in our cases.

Among the variables presented in Tables 1 and 2 , our study included patients who only received immediate prepectoral DTI. Patients who had already received partial mastectomy or prior radiation therapy before total mastectomy were excluded. This enrollment scheme eliminated bias caused by radiation-induced capsule fibrosis, poor quality of breast skin, and tissue atrophy. Nipple-sparing mastectomy was the most common mastectomy type (84.9%), and a periareolar with lateral extension incision approach was mainly used. This is suitable for pectoralis major muscle access for the superior coverage technique. However, if the oncologic surgeon prefers an inframammary fold incision when performing mastectomy, then the superior coverage technique may be difficult to implement due to a poor visual field.

The risk of rippling is dependent on numerous extrinsic and intrinsic factors. 23 Multivariable regression analysis was performed to control multiple possible confounding variables. According to Tables 3 and 4 , rippling was significantly less common in patients with high BMI, concurrent with results in previous studies assessing prepectoral implant-based breast reconstruction. This result can be attributed to the high BMI, which increases the probability of thicker remnant subcutaneous tissue that can better cover the implant, lessening rippling. In the same context, the greater is the subcutaneous fat preservation, the less likely is rippling may be. Quantitative measurements of subcutaneous thickness using ultrasonic devices and a comparison of the subcutaneous tissue preservation effect based on the breast surgeon's preferred methods would be good focal areas of further study.

There was significantly less rippling in patients with an adjuvant chemotherapy history among pre- and postoperative oncologic therapies in both the univariate and multivariate analyses. Second- or third-generation taxane-based regimens (paclitaxel, docetaxel) have been widely used as adjuvant systemic therapies in breast cancer patients. 24 Fluid retention, which includes peripheral edema and sometimes associated with weight gain, is a side effect of docetaxel. 25 26 27 28 It is also known that taxane-based chemotherapy regimens can cause lymphedema. 29 Based on these studies, it may be possible to decrease the severity of rippling by masking the edematous state of the breast upper pole skin flap. However, further studies are needed to support this hypothesis with a better understanding of the pathophysiology of anticancer drug side effects.

The two types of implants used in this study have different filling rates and cohesiveness. According to previous studies, 30 31 the rippling rate was high in implants with low cohesivity, but this study revealed no significant differences in rippling according to implant type. 30 Since there was no significant difference in cohesiveness between the two implants, it is unlikely that there was a significant difference in our study. Assuming that rippling is dependent on the thickness of the remaining breast tissue, ADMs from three companies with two thicknesses were compared, but no significant difference was observed. This suggests that the use of ADM may not eliminate rippling completely, as shown in previous studies. 18

Notably, in both the univariable and multivariable regression analyses, rippling was significantly less common in patients who underwent breast reconstruction using the superior coverage technique. Propensity score matching was performed by including all variables that could affect rippling as much as possible and randomized matching with the same number of people could be appropriate for the two experimental groups ( Table 5 ). In both groups, conditional logistic regression with rippling as the dependent variable was performed, and a significant *p* -value (0.017) was attained ( Table 6 ). After propensity score matching, the identity document of each group was matched to conduct conditional logistic regression by considering the matching information between them. Through various analyses, the factors influencing rippling were analyzed, and this may be meaningful given that it confirms the significance of the superior coverage technique.

There are several limitations to this study given its retrospective nature, single-center design, and limited follow-up period. Unlike previous studies assessing the superior coverage technique, our comparison with a control group and randomizing participants through matching increased the reliability of this study, but there may have been bias left due to the relatively small sample size. Also, determining rippling was a subjective criterion even though patients were evaluated by an expert. Despite these limitations, this study can be considered meaningful given its improvements to randomize various statistical methods and variables. A prospective and multi-institutional study with a longer follow-up period is planned to obtain additional evidence supporting the superior coverage technique in implant breast reconstruction procedures.

In patients undergoing prepectoral DTI breast reconstruction and implant-based breast reconstruction, rippling continues to occur, without a clear solution. According to our study, breast implant rippling was significantly reduced in patients with higher BMI, postoperative chemotherapy, and superior coverage technique. We propose a modified superior coverage technique that can significantly reduce rippling. Additional studies are required to explore the effects of these methods in prepectoral prosthetic-based breast reconstruction.
